# Genome Editing Technology and Its Application to Metabolic Engineering in Rice

**DOI:** 10.1186/s12284-022-00566-4

**Published:** 2022-04-02

**Authors:** Satoru Sukegawa, Seiichi Toki, Hiroaki Saika

**Affiliations:** 1 Institute of Agrobiological Sciences, National Agriculture and Food Research Organization, 3-1-3 Kannondai, Tsukuba, Ibaraki 305-8604 Japan; 2 Graduate School of Nanobioscience, Yokohama City University, Yokohama, Kanagawa Japan; 3 Kihara Institute for Biological Research, Yokohama City University, Yokohama, Kanagawa Japan; 4 Department of Plant Life Science, Faculty of Agriculture, Ryukoku University, Otsu, Shiga Japan

**Keywords:** Genome editing, Molecular breeding, Metabolic engineering

## Abstract

Genome editing technology can be used for gene engineering in many organisms. A target metabolite can be fortified by the knockout and modification of target genes encoding enzymes involved in catabolic and biosynthesis pathways, respectively, via genome editing technology. Genome editing is also applied to genes encoding proteins other than enzymes, such as chaperones and transporters. There are many reports of such metabolic engineering using genome editing technology in rice. Genome editing is used not only for site-directed mutagenesis such as the substitution of a single base in a target gene but also for random mutagenesis at a targeted region. The latter enables the creation of novel genetic alleles in a target gene. Recently, genome editing technology has been applied to random mutagenesis in a targeted gene and its promoter region in rice, enabling the screening of plants with a desirable trait from these mutants. Moreover, the expression level of a target gene can be artificially regulated by a combination of genome editing tools such as catalytically inactivated Cas protein with transcription activator or repressor. This approach could be useful for metabolic engineering, although expression cassettes for inactivated Cas fused to a transcriptional activator or repressor should be stably transformed into the rice genome. Thus, the rapid development of genome editing technology has been expanding the scope of molecular breeding including metabolic engineering. In this paper, we review the current status of genome editing technology and its application to metabolic engineering in rice.

## Main Text

### Current Status of Genome Editing Technology in Rice

**Fig. 1:**
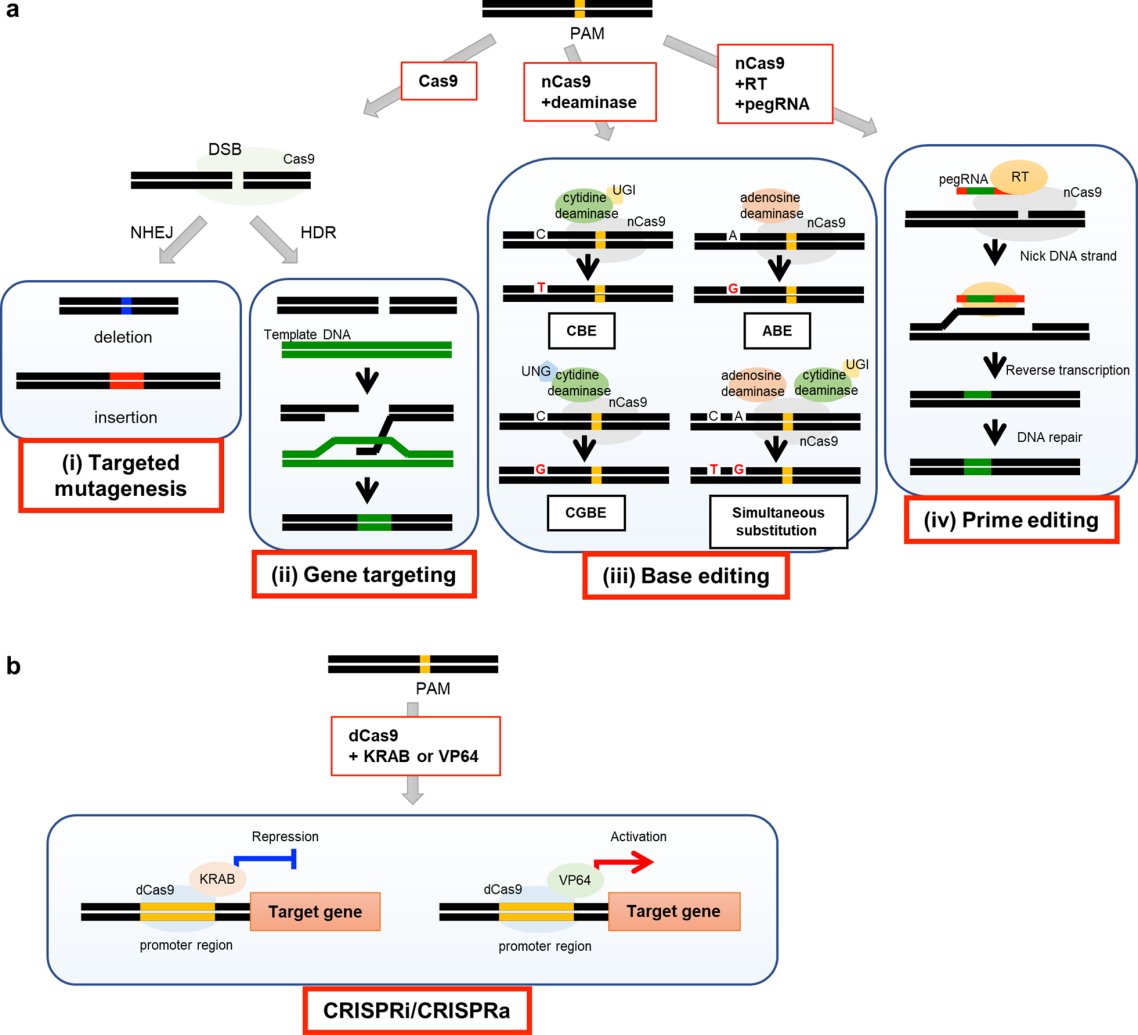
Summary of latest genome editing technologies. **a** Genome editing technologies involved in modification of DNA sequences. (i) Cas9 delivered to the target sequence by gRNA causes targeted mutagenesis. (ii) If template DNA is present, GT occurs, but infrequently. (iii) Base substitution can be introduced into a target sequence by a base editor. (iv) By combining nCas9 with pegRNA and RT, the target sequence can be inserted accurately into the target site: “prime editing”. **b** Genome editing without modification of DNA sequence. CRISPRi/CRISPRa can regulate gene expression of a target gene using dCas9 and a transcription factor such as KRAB or VP64. See main text for details of each technology

Metabolic engineering can be used to improve rice quality, for example via fortification of nutrients or reduction of unhealthy compounds. The artificial modification of genes involved in the metabolism of a target compound enables regulation of the content of that compound. Recent progress in genome editing technology to allow precise modification of a target sequence will help with breeding such useful rice varieties (Fig. 1a). Here, we will first summarize and illustrate recent trends in the genome editing technology used in rice.

#### Targeted Mutagenesis

Targeted mutagenesis describes the technology used to introduce mutation by induction of DNA double-strand breaks (DSBs) in a target sequence and subsequent endogenous DNA repair mechanisms. DSBs are introduced using artificial nucleases such as Transcription activator-like effector (TALE) nucleases (TALENs) and clustered regularly interspaced short palindromic repeat (CRISPR)/CRISPR-associated protein (Cas). Targeted mutagenesis often results in the insertion or deletion of a few bases at the cleavage site by artificial nucleases and is therefore commonly used to disrupt target genes. In rice, gene disruption by targeted mutagenesis and accompanying improvement of agronomical traits has been reported. Among CRISPR/Cas systems, CRISPR/Cas9—consisting of Cas9 nuclease and a guide RNA (gRNA) targeting the sequence of interest—is used most widely, especially SpCas9 derived from *Streptococcus pyogenes*. Li et al. (2016) mutated *OsGN1a*, *OsDEP1*, and *OsGS3* using the CRISPR/Cas9 system and these mutants showed an increase in grain number, dense and erect panicles, and an increase in grain size, respectively. Another report by Lv et al. (2021) showed that knocking out *OsPAO5*, which encodes polyamine oxidases in rice, using CRISPR/Cas9 can markedly increase grain length, 1000-g weight, grain number per panicle, and yield per plant. Moreover, the expression of multiple gRNAs targeting different genes enables simultaneously targeted mutagenesis of target genes. Cas nuclease uses the protospacer adjacent motif (PAM) sequence as a clue to recognize and cleave the target DNA. The PAM sequence differs depending on the type of Cas nuclease, and for SpCas9 PAM sequence is 5′-NGG-3′. This is one of the limitations to the selectivity of target sequences. Cas nucleases other than Cas9 have also been developed as tools for genome editing to address this problem; the CRISPR/Cas system has a wide diversity in nature, with many different and unique PAM sequences. Cas12a and Cas12b, which recognize a T-rich PAM, have also been applied to crop genome editing and succeeded in introducing mutations in endogenous rice genes (Endo et al. 2016; Ming et al. 2020; Tang et al. 2017b). Cas proteins other than Cas9, Cas12a, and Cas12b have not yet been exploited in rice genome editing. TiD (Type I-D) and Cas3, belonging to the Class 1 CRISPR system, have been reported as new genome editing systems (Morisaka et al. 2019; Osakabe et al. 2020; 2021). Unlike Cas9 and Cas12, Class 1 CRISPR system comprise multiple Cas proteins known as CRISPR-associated complex for antiviral defense (Cascade) as well as nucleases (Cas10d and Cas3, respectively). When mutations were introduced into human cells and tomatoes using TiD, small deletions of several bases or large bi-directional deletions of several kilobases were confirmed (Osakabe et al. 2020), although Cas3 has been shown to induce uni-directional large deletion in human cells (Morisaka et al. 2019), as yet there has been no report of the application of Cas3 to genome editing in plants. In addition, it has been reported that Cas12f, a miniature size Cas that is nearly half the size of Cas9 and Cas12a, successfully introduced mutations in bacteria, human cells, and maize (Bigelyte et al. 2021; Wu et al. 2021; Xu et al. 2021a) and is expected to be applied to genome editing in rice. Thus, there are many Cas nucleases that can be used in rice. With their different specific properties, such as protein size, PAM sequence, and mutation spectrum, Cas nucleases could be used for different purposes according to the specific application required.

#### Base Editing

Base editing (BE) is a useful technology capable of substituting an amino acid sequence, inducing functional modification of a target protein. The nuclease domains in Cas9 (RuvC and HNH) introduce a nick in each DNA strand, resulting in DSBs. The BE strategies developed so far utilize a fusion protein of nickase type Cas9 (nCas9) which is deactivated RuvC domain or dead Cas9 (dCas9) which is deactivated RuvC and HNH domain together with a base-modifying enzyme. The cytosine base editor (CBE) system converts the target base from C to T by combining nCas9 and cytidine deaminase. The base substitution activity is reported to be increased by binding uracil DNA glycosylase inhibitor (UGI) to the C-terminus of CBE (Komor et al. 2016; Nishida et al. 2016). On the other hand, the adenine base editor (ABE) can induce base substitution from A to G by binding adenosine deaminase to nCas9 (Gaudelli et al. 2017). Both these BE technologies can be applied to rice (Molla et al. 2021). When using target-activation-induced cytidine deaminase (target-AID), with a fusion protein including nCas9 fused to *Petromyzon marinus* cytidine deaminase (PmCDA1) and gRNAs for two target sites, *OsFTIP1e* and *OsALS* simultaneous C to T substitutions in multiple regions were enabled (Shimatani et al. 2017). Moreover, an A to G substitution was introduced in the *OsACC* encoding acetyl-coenzyme A carboxylase by using ABE7.10—a fusion of an adenosine deaminase (ecTadA-ecTadA*) with nCas9 and gRNA (Li et al. 2018). Plants modified using BE showed herbicide resistance (Li et al. 2018; Shimatani et al. 2017). More recently, the simultaneous introduction of base substitutions (C to T and A to G) by fusing both CBE and ABE to nCas9 was reported (Li et al. 2020; Sakata et al. 2020). The group of Li et al. (2020) developed Saturated Targeted Endogenous Mutagenesis Editors (STEMEs), i.e., proteins that fuse nCas9 and human APOBEC3A (as a cytidine deaminase with wide deamination window activity, as shown below) and UGI for C to T substitution and TadA (as adenosine deaminase) for A to G substitution, and succeeded in inducing efficient base substitution (C to T and A to G) in rice. In addition, by applying Target-ACEmax, which is a fusion of PmCDA1 and UGI as a cytidine deaminase and TadA as an adenosine deaminase, to nCas in cultured human cells, the simultaneous introduction of base substitutions (C to T and A to G) was achieved (Sakata et al. 2020).

The window in which base substitution can be introduced by BEs differs and is dependent on the base substitution enzyme, e.g., in rat APOBEC1, the window is 17–12 bases upstream of the PAM sequence (Zong et al. 2017), in human APOBEC3A, it is 20–4 bases upstream of the PAM sequence (Zong et al. 2018), and in ABE7.10, it is 17–12 bases upstream of the PAM sequence (Kang et al. 2018). Given these limitations in the window and PAM sequence, it is not always possible to introduce a base substitution precisely at a target base. One way to solve this problem is to use other Cas nucleases, such as the Cas9 derived from species other than *S. pyogenes*, (e.g. SaCas9, *Staphylococcus aureus*-derived Cas9 whose PAM is 5'-NNGRRT-3', and Cas12a, which recognizes a T-rich PAM (5'-TTTN-3')) or by using a Cas that can modify PAM recognition, such as SpCas9-NG, which recognizes 5'-NG-3' as a PAM sequence, by improving the amino acid sequence of the PAM recognition site of SpCas9 (Nishimasu et al. 2018). Using the former method, Qin et al. (2019) reported the C to T or A to G substitution in rice using CBE or ABE, whereas, using the latter approach, Endo et al. (2019b) and Negishi et al. (2019) succeeded in inducing C to T and A to G transitions in target genes of rice by using nSpCas9-NGv1 (a variant of SpCas9-NG)-based target-AID, and ABE7.10, respectively. SpCas9-NG can also be applied to the simultaneous substitution of C to T and A to G similar to SpCas9. In addition, BEs using other PAM-modified SpCas9, such as xCas9 and SpRY (Hu et al. 2018; Walton et al. 2020), have been applied successfully to plants including rice (Ren et al. 2021; Xu et al. 2021c; Zhong et al. 2019).

More recently, C to G and C to A substitutions in *Escherichia coli* (Zhao et al. 2021) and human cells (Kurt et al. 2021) using cytidine deaminase and uracil DNA glycosylase were reported. The successful introduction of C to G substitutions using cytidine deaminase and uracil DNA glycosylase in plants including rice, showing that C to G substitution can also be applied to plants (Sretenovic et al. 2021). In principle, all types of substitution are possible by applying the BEs, CBE, ABE for C to G or A; for example, A to C substitution is achieved by A·T to G·C by ABE, with subsequent C·G to A·T and A·T to G·C by CBE in *E. coli* (Zhao et al. 2021). However, universal BE in one step has not yet been developed.

BEs are precise genome editing tools because they enable the introduction of a substitution at a target base. However, their windows are not always narrow, as noted above, resulting in the occurrence of “bystander” mutations. Taking advantage of this point, some groups have reported targeted random mutagenesis using BEs in the specific region of a target gene in rice. Li et al. (2020) found a novel mutation conferring herbicide tolerance by screening herbicide-tolerant plants from pools of mutants produced by STEMEs. They focused on the carboxyltransferase domain of rice ACC, which is the target of herbicides such as haloxyfop (Powles and Yu 2010). Using STEMEs, substitutions resulting in silent, missense, and nonsense mutations were introduced in a total of 209 amino acids out of targeted 400 amino acids in the carboxyltransferase domain (Li et al. 2020). Interestingly, a plant harboring *OsACC* with a novel substitution to confer haloxyfop resistance was successfully screened, demonstrating targeted random mutagenesis using BEs.

#### Precise Genome Editing Using a Donor DNA/RNA

Gene targeting (GT) is a precise modification method using donor DNA which is a template to repair DNA DSBs. An efficient selection method of cells that have succeeded in GT is necessary because the frequency of GT occurrence is much lower than the random integration of donor DNA into the host genome in flowering plants (Shimatani et al. 2015). When mutations to confer drug resistance are introduced into a target gene by GT, cells that succeed in GT show drug resistance and can be selected using a herbicide or an amino acid analog (Endo et al. 2006, 2007; Hanin et al. 2001; Saika et al. 2011). In addition, a positive–negative selection method that can be applied, in principle, to the modification of any target gene was developed in mouse embryo-derived stem cells (Mansour et al. 1988). In this method, cells in which the target sequence has been incorporated can be enriched efficiently using a positive selection marker to select cells that have succeeded in GT and a negative selection marker that suppresses the growth of cells in which the GT vector was inserted at a random position. A knockout of the *OsWx* in rice by insertion of a positive selection marker is the first report of GT using positive–negative selection in plants (Terada et al. 2002). By combining selection marker removal methods using Cre/*lox*P, *piggyBac* transposon, or single-strand annealing, it has become possible to remove a positive selection marker to leave only the desired mutation in the target gene (Dang et al. 2013; Endo et al. 2020; Nishizawa-Yokoi et al. 2015; Ohtsuki et al. 2020; Terada et al. 2010).

In rice, several methods have been reported to increase the frequency of GT for breeding applications. One of the factors limiting GT efficiency is the infrequent delivery of a donor DNA for GT to plant cells. An *in planta* GT method was reported in Arabidopsis (Fauser et al. 2012). In this method, a donor DNA is pre-inserted into the genome of host plant and GT is induced when required. Thus, this method avoids dependence on the frequency of donor DNA delivery. Nishizawa-Yokoi et al. (2020) applied *in planta* GT to rice by using an all-in-one vector harboring a CRISPR/Cas9 expression cassette, a selectable marker, and a GT donor. Moreover, another method for improving GT efficiency using the reagent Rad51-stimulatory compound 1 (RS-1), which improves the frequency of homologous recombination, has been reported (Jayathilaka et al. 2008). Nishizawa-Yokoi et al. (2020) showed that, when inducing two amino acid substitutions into *OsALS* by GT, the efficiency of GT was increased slightly in callus pre-cultured for 2 weeks in selective medium containing RS-1.

Although gene modifications using donor RNA as a template have been reported in rice (Butt et al. 2017; Li et al. 2019), more recently prime editing (PE), a new precise genome editing method using RNA as a template, has also been reported. Anzalone et al. (2019) reported a PE system using a prime editing gRNA (pegRNA) in which reverse transcriptase (RT) was fused to nCas9 and an RNA sequence homologous to the target DNA was ligated to the gRNA. In this technique, the nCas9 delivered to the target region by pegRNA first nicks the target DNA strand. Next, the pegRNA binds the nicked DNA, and the RT fused to nCas9 synthesizes a DNA strand using the pegRNA target sequence as a template. The synthesized target DNA is then inserted into the target sequence to complete GT. The advantage of PE is that off-target activity, as seen with Cas9, is extremely low because PE induces no DNA DSBs, and it is a very useful technique in situations where more accurate editing is required. PE applied to rice and wheat enabled introduction of insertions, deletions or point mutations in the endogenous targeted gene on a case by case basis (Lin et al. 2020). Lin et al. (2021) developed a more efficient PE method using two pegRNAs to encode the same edits in the forward and reverse direction of each DNA strand simultaneously (dual-pegRNA strategy); PE efficiency was enhanced when this system was applied to rice. Suppression of mismatch repair pathway has been reported to improve PE frequency in human and mouse cells (Chen et al. 2021), and thus could be applied to rice to enhance PE frequency.

#### CRISPRi and CRISPRa

Gene expression is regulated by the binding of transcriptional regulators such as transcription factors (TFs) to the promoter region of a gene. CRISPRi and CRISPRa (Fig. 1b) regulate endogenous gene expression using the CRISPR/Cas system, fusion proteins including dCas9 and transcriptional repression domains (such as KRAB, Krüppel-associated box domain or SID4X, four concatenated mSin3 interaction domains) or activation domains (VP64 or p65 subunit of nuclear factor kappa B) (Gilbert et al. 2013; Konermann et al. 2013; Sander and Joung 2014). Chavez et al. (2015) developed more efficient Cas9 transcriptional activator systems fusing multiple transcriptional activation domains (VP64-p65-Rta) to dCas9, and confirmed a significant increase in endogenous gene expression compared with fusing a single activator when this system applied in human cells. This technology can regulate gene expression in *E. coli* (Bikard et al. 2013), human cells (Cheng et al. 2013; Qi et al. 2013), and yeast (Smith et al. 2016). Several applications of CRISPRi/CRISPRa have been reported [refer to reviews such as Pan et al. (2021)]. For example, Li et al. (2017) modified the transcriptional activation domain to enable efficient use of the CRISPRa system in plants by constructing dCas9-TV (dCas9-6xTAL-VP128) and applying it to Arabidopsis or rice. The expression of target genes was increased to levels dozens of times that of baseline (Li et al. 2017). CRISPRi/CRISPRa are thus useful tools with which to regulate the expression of target proteins, including enzymes involved in metabolic pathways, although CRISPRi/CRISPRa expression vectors should be stably introduced into the rice genome.

### Improvement of Grain Components by Genome Editing

**Fig. 2:**
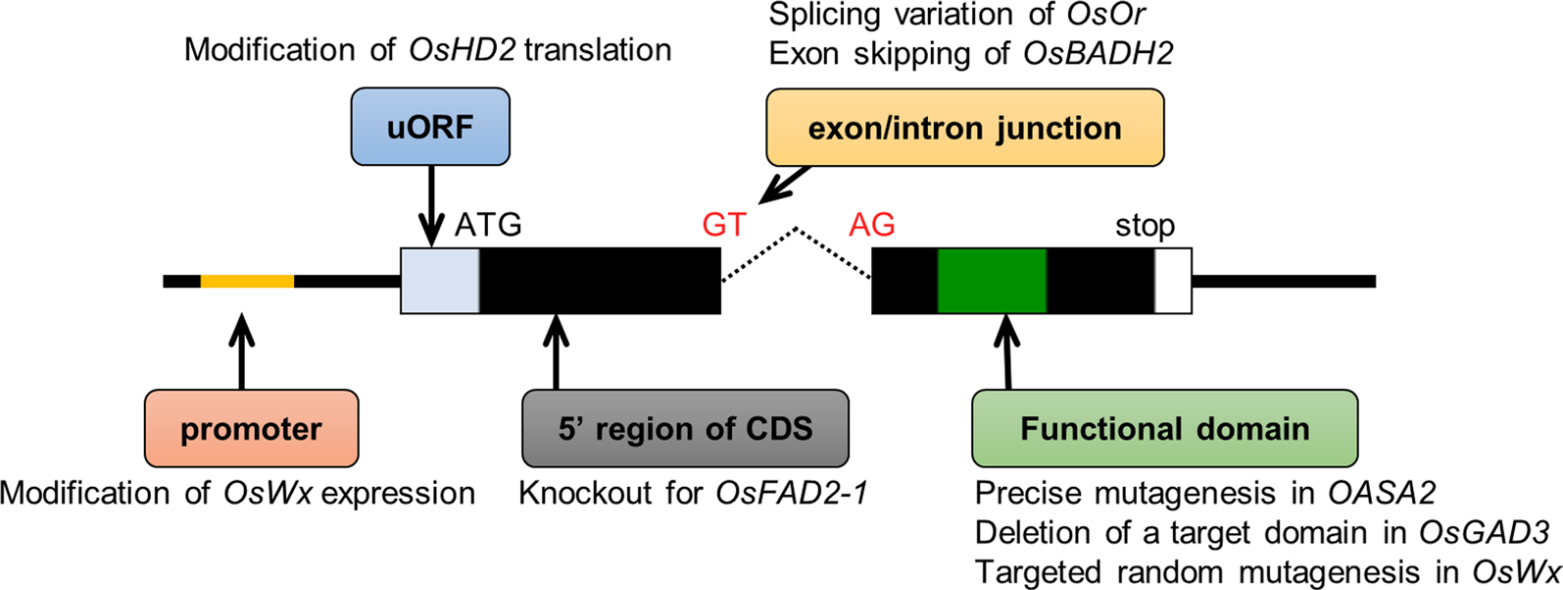
Target regions of gene modification by genome editing technology. Regions targeted by genome editing and examples of gene modification shown in this review are summarized. The yellow line indicates the cis-regulatory element in the promoter region of a target gene. Light blue and white boxes indicate 5′ and 3′ untranslated regions of a target gene, respectively. Black and green boxes indicate coding regions of a target gene and a region corresponding to the functional domain of a translated protein, respectively. Solid and dotted lines indicate the target gene locus and the intron of the target gene, respectively

Enhancement of a biosynthetic pathway and/or suppression of a catabolic pathway can be used to fortify the amount of a specific metabolite. Inactivation of a target enzyme is the simplest case of metabolic engineering using genome editing technology and has been employed widely. In addition, the inactivation of a target enzyme enables fortification of a metabolite into which a substrate of the target enzyme is catabolized. In general, the enhancement of biosynthetic enzymes is more difficult because gain-of-function mutations need to be introduced precisely into the target enzyme. These approaches are efficient for metabolic engineering in cases where the target gene to be modified by genome editing and mutations to improve a target trait are already clear. However, in some cases, there is little information on which mutations should be introduced into a target gene to confer a desirable trait, even if genes encoding target enzymes involved in a particular metabolic pathway are known. In such cases, an approach to produce mutants with a diverse range of phenotypes can be applied. In addition to the above, modification of the expression levels of a target gene can be another approach to the production of mutants with a diverse range of phenotypes. For example, Rodríguez-Leal et al. (2017) produced numerous variants in the promoter of a target gene by CRISPR-Cas9-mediated genome editing in tomato and succeeded in the modification of traits such as fruit size, inflorescence branching, and plant architecture. Here, we show some examples of metabolic engineering using genome editing (Fig. 2). Please refer to other reviews (e.g., Zafar et al. (2020)) for examples not shown here.

#### Amylose content

A granule-bound starch synthase (GBSS) encoded by *Wx* is involved in amylose synthesis in plants. Inactivation of GBSS results in lower amylose contents in rice grain. For example, Terada et al. (2002) reported the reduction of amylose content by knockout of *Wx* by GT and Nishizawa-Yokoi et al. (2016) reported targeted mutagenesis using TALENs. On the other hand, Xu et al. (2021b) produced several genome-edited rice plants harboring GBSS with novel amino acid substitutions at target regions by CBE. Some mutants showed a slight decrease in the apparent amylose grain content, with some mutants decreased significantly (Xu et al. 2021b), suggesting that this approach can be applied to produce mutants with a diverse range of phenotypes. Furthermore, Zeng et al. (2020) used a CRISPR/Cas9 system to produce mutations in putative cis-regulatory elements in the promoter region of *Wx*. Some mutants showed a decrease in *Wx* expression levels in developing endosperm and lower amylose content (Zeng et al. 2020). Thus, modification of GBSS activity and *Wx* gene expression led to a slight reduction in amylose content.

#### Amino acid content

Gamma-aminobutyric acid (GABA) is known as a health-promoting amino acid. Akama et al. (2020) succeeded in the production of GABA-fortified rice by CRISPR-Cas9-mediated targeted mutagenesis of *OsGAD* encoding a glutamate decarboxylase (GAD), which catalyzes the conversion from glutamate to GABA, and has an autoinhibitory domain at its C-terminus. They focused on *OsGAD3*, which is expressed predominantly in rice seeds, and showed that truncated OsGAD3 had higher enzymatic activity in vitro (Akama et al. 2020). They succeeded in the production of GABA-fortified rice by precise deletion using two gRNAs designed to remove the autoinhibitory region (Akama et al. 2020). Genome-edited tomato harboring truncated GAD lacking the autoinhibitory domain accumulated GABA in its fruit (Nonaka et al. 2017), suggesting that this strategy for GABA fortification could be applied widely to other crops.

A successful example of metabolic engineering by precise mutagenesis via GT in rice is that of anthranilate synthase. A key enzyme in the tryptophan biosynthesis pathway, the enzymatic activity of anthranilate synthase is controlled by feedback regulation of tryptophan (Wakasa and Ishihara 2009). Mutations modifying enzymatic properties, such as enhancement of catalytic activity and/or increase in insensitivity to tryptophan, have been reported (Kanno et al. 2005) and were introduced into *OASA2*, which encodes a subunit of anthranilate synthase, via GT (Saika et al. 2011). As expected, free tryptophan accumulated highly in seedlings and seeds of GT rice plants (Saika et al. 2011). In this case, in vitro assay had already shown that substitutions of amino acid residues in OASA2 improved enzymatic properties (Kanno et al. 2005). The information obtained from structure-based protein engineering is useful for rice trait improvement via genome editing. Moreover, the release of feedback regulation via precise gene modification could be applied to modify the contents of amino acids other than tryptophan.

#### Fatty acid composition

Oleic acid is oxidatively stable and helps suppress lifestyle diseases (Lopez-Huertas 2010) so high oleic acid oil is an important breeding target in oil crops such as soybean. *FAD2* encodes fatty acid desaturase—an enzyme converting oleic acid into linoleic acid in plants. Many previous reports of knockdown and knockout of *FAD2* in plants have been published, e.g., knockdown of *FAD2* by RNA interference (RNAi) and knockout of *FAD2* by TALENs and CRISPR/Cas9-mediated targeted mutagenesis in soybean (Demorest et al. 2016; Haun et al. 2014). In rice, knockdown mutants of *OsFAD2-1*—the highest expressing *OsFAD2* homologs in rice seeds*—*showed higher oleic acid content (Zaplin et al. 2013). Abe et al. (2018) produced knockout plants of *OsFAD2-1* by CRISPR-Cas9 mediated targeted mutagenesis. The oleic acid content of brown rice in *OsFAD2-1* knockout plants increased to more than twice that of wild type (Abe et al. 2018).

#### β-carotene

Biofortification of β-carotene (provitamin A) is one of the important breeding targets in crops. *Orange* (*Or*), which encodes a protein chaperone, is involved in carotenoid biosynthesis, and expression of splicing variants of *Or* enhances carotenoid accumulation in cauliflower (Lu et al. 2006). Based on information on splicing variants of *Or* transcripts reported in cauliflower, Endo et al. (2019a) succeeded in the fortification of carotenoid in rice calli by expressing splicing variants of *OsOr* through the artificial introduction of mutations at the splicing donor site of *OsOr*. Moreover, Dong et al. (2020) successfully created a knock-in of 5.2-kb of an expression cassette of carotenoid biosynthesis genes into a targeted locus of rice. β-carotene accumulated to a high level in the endosperm of knock-in rice (Dong et al. 2020).

#### Fragrance

2-Acetyl-1-pyrroline (2AP) is a major volatile compound responsible for the fragrance of aromatic rice. Conversion of gamma-aminobutyraldehyde to GABA is catalyzed by rice betaine aldehyde dehydrogenase, encoded by *OsBADH2* (Bradbury et al. 2008; Chen et al. 2008). If OsBADH is inactivated, gamma-aminobutyraldehyde is converted to delta-1-pyrroline and subsequently to 2AP (Bradbury et al. 2008; Chen et al. 2008). The 2AP content in grain was increased by knockout of *OsBADH2* by TALENs-mediated targeted mutagenesis of the 4th exon (Shan et al. 2015), and 2nd exon skipping was caused by a 1- or 3-bp deletion at the junction of the 2nd exon and the intron (Tang et al. 2021). In addition, simultaneously targeted mutagenesis of three cytochrome P450 genes and *OsBADH2* conferred both increased grain size and higher 2AP content (Usman et al. 2020).

#### Cadmium

Transporter can be targets to fortify or reduce a specific component such as a micronutrient. The concentration of cadmium, which is toxic to humans, in brown rice could be reduced dramatically by the knockout of *OsNramp5*, which is involved in the uptake of cadmium by roots (Tang et al. 2017a). In this case, information obtained from basic research in rice (Ishikawa et al. 2012; Sasaki et al. 2012) can be applied to the breeding of extensively used *indica* parental lines of hybrid rice.

## Prospects

In this review, we have summarized the current status of genome editing technologies in plants including rice, and shown examples of metabolic engineering via genome editing in rice. Genome editing technology is developing at an ever-increasing pace in various organisms, and can often be applied to rice immediately. Many reports of trait improvement, including metabolic engineering via genome editing, in addition to the examples introduced in this review, have been published in rice. Besides the technologies shown above, organellar genome editing, such as the large deletion of mitochondria-encoded genes by TALENs with a mitochondria localization signal (Kazama et al. 2019), and C to T substitution in chloroplast-encoded *psaA* by cytidine deaminase and UGI fused to TALEs with a chloroplast localization signal (Li et al. 2021) have been reported in rice. Organellar genome engineering could be also applied to metabolic engineering in rice. Moreover, Lu et al. (2021) reported the successful promoter swap via targeted inversion and duplication of 911-kb and 338-kb regions, respectively. Taken together, all these genome editing strategies represent one candidate technology that can be used widely for rice trait improvement.

However, due to the intrinsically complex nature of metabolic pathway networks, metabolic engineering is not always easy. To date, several approaches to metabolic engineering by knock-out of multiple genes have been reported. For example, targeted mutagenesis of *GmF3H1*, *GmF3H2* and *GmFNSII‐1* (encoding enzymes involved in bypass pathway of isoflavone biosynthesis) resulted in higher isoflavone content in mutant leaves in soybean (Zhang et al. 2020). Moreover, fine-tuning of target metabolite content in rice grains may allow the precise modification of the many enzymes involved in biosynthesis and catabolic pathways. In addition, regulation of the expression levels and patterns of target gene expression would be required. In rice, translation of *OsHD2* could be enhanced by targeted mutagenesis in the upstream open reading frames (uORFs) located in the 5′ upstream region of the primary ORF to control translation initiation at its primary ORF (Liu et al. 2021). Genome editing in *cis*-regulatory elements and the uORF could be a candidate tool for metabolic engineering also. Gene expression might be controlled more dramatically by modification of both the promoter and uORF of the target gene. In yeast, the combination of gene knockout by CRISPR-Cas9, and modification of gene expression by CRISPRa and CRISPRi was applied successfully to rational metabolic engineering (Lian et al. 2017). Thus, the simultaneous modification of many genes via various types of genome editing, gene knockout, precise substitution, and enhancement and/or suppression of gene expression can also be applied to metabolic engineering in rice (Pan et al. 2021). To achieve this, genome editing technology, and related technology such as delivery systems for getting genome editing tools into plant cells, need to be improved universally and efficiency increased to meet demand. Moreover, information on the target genes and ideal genotypes is also essential. Much more omics data and genome design systems based on such data to optimize target metabolite content will be necessary for further metabolic engineering. Analysis of multi-omics data with a machine learning approach could provide us with ideal genotypes and metabolic pathways. It has been proposed that integration of systems biology and machine learning will enhance metabolic engineering, especially in microorganisms (Helmy et al. 2020). With subsequent rapid transfer of these new technologies to other organisms, the future of genome editing and metabolic engineering in rice looks bright.

## Data Availability

Not applicable.

## Abbreviations

2AP: 2-Acetyl-1-pyrroline
ABE: Adenine base editor
BE: Base editing
Cas9: CRISPR-associated protein 9
Cascade: CRISPR associated complex for antiviral defense
CBE: Cytosine base editor
CRISPR: Clustered regularly interspaced short palindromic repeat
dCas9: Dead Cas9
DSBs: Double-strand breaks
GABA: Gamma-aminobutyric acid
GAD: Glutamate decarboxylase
GBSS: Granule-bound starch synthase
gRNA: Guide RNA
GT: Gene targeting
nCas9: Nickase Cas9
Or: Orange
PAM: Protospacer adjacent motif
PE: Prime editing
pegRNA: Prime editing gRNA
PmCDA1: *Petromyzon marinus* Cytidine deaminase
RNAi: RNA interference
RS-1: Rad51-stimulatorycompound 1
RT: Reverse transcriptase
STEMEs: Saturated Targeted Endogenous Mutagenesis Editors
TALE: Transcription activator-like effector
TALENs: TALE nucleases
target-AID: Target-activation-induced cytidine deaminase
TFs: Transcription factors
TiD: Type I-D
UGI: Uracil DNA glycosylase inhibitor
uORFs: Upstream open reading frames
